# Tunable Nanoscale Metal‒Molecule‒Semiconductor Junctions via Light‐Controlled Molecular Orientation

**DOI:** 10.1002/smll.202412438

**Published:** 2025-05-19

**Authors:** Essam Mohamed Dief, Tiexin Li, Ingrid Ponce, Nadim Darwish

**Affiliations:** ^1^ School of Molecular and Life Sciences Curtin University Bentley WA 6102 Australia; ^2^ Departamento de Ciencias del Ambiente Facultad de Química y Biología Universidad de Santiago de Chile Av. Libertador Bernardo O'Higgins 3363, Estación Central Santiago 9170022 Chile

**Keywords:** metal‒semiconductor junctions, molecular electronics, molecular switches, molecule‒electrode contacts, silicon

## Abstract

Molecular electronics offers significant potential for the development of miniaturized and tunable electronic devices, with silicon (Si) remaining a cornerstone of modern semiconductor technology. This study presents a method for constructing tunable molecular circuits on Si electrodes using UV‐controlled hydrosilylation reaction, enabling precise control over molecular orientation and bonding. When hydrogen‐terminated Si surfaces are exposed to UV light in a solution of 9‐decyne‐1‐ol, hydroxyl (OH) groups form covalent Si─O─C bonds, while in the absence of UV light, alkyne groups instead react to form Si─C bonds. This tunability allows precise positioning of oxygen atoms near the Si surface, thereby enhancing charge transfer in metal–molecule–semiconductor junctions and at electrified semiconductor–electrolyte interfaces. Conducting atomic force microscopy (C‐AFM) measurements reveal that Pt─Si junctions exhibit Schottky diode characteristics, whereas Pt–molecule–Si junctions display Ohmic behaviour. Junctions formed via Si─O bonds demonstrate significantly lower resistance and at least a two‐fold higher electron transfer rate constant (*k*_et_) compared to those formed with Si─C bonds, indicating superior charge transfer when oxygen atoms are positioned near the Si electrode. These findings suggest that incorporating oxygen‐containing molecules reduces the space‐charge region, thereby facilitating current flow at the Si–metal and Si–electrolyte electrified interfaces.

## Graphical abstract

This study demonstrates tunable molecular circuits on silicon electrodes through UV‐controlled surface reactions involving hydroxyl and alkyne groups, enabling precise formation of Si─O─C or Si─C bonds. The proximity of oxygen atoms to the silicon surface enhances charge transfer, resulting in lower resistance and faster electron transfer kinetics. These findings advance the development of molecular–silicon hybrid electronics.

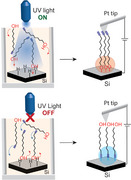

## 1 Introduction

Molecular electronics has emerged as a promising approach for developing next‐generation electronic devices, enabling miniaturization and tunability at the molecular scale. Integrating molecular circuits into silicon‐based electronics is particularly compelling, as silicon remains the foundation of the modern semiconductor industry.^[^ ^1^, ^2^, ^3^, ^4^, ^5^, ^6^ ^]^ The ability to construct molecular junctions on silicon surfaces opens pathways for bridging the gap between molecular electronics—predominantly based on metal electrodes—and the well‐established silicon microelectronics industry. Unlike conventional molecular circuits that rely on metal electrodes,^[^ ^7^, ^8^, ^9^, ^10^, ^11^, ^12^, ^13^ ^]^ silicon offer a broader range of interfacial bonding while still maintaining their semiconducting properties. This integration enables the development of functional devices such as transistors, diodes, and more complex electronic components, with capabilities that exceed those achievable by molecular electronics or semiconductor systems separately.

A key aspect of molecular functionalization is its influence on the space charge region (also known as the depletion region) at the interface between silicon and metals or between silicon and electrolytes in electrochemical measurements.^[^ ^14^, ^15^, ^16^, ^17^, ^18^, ^19^, ^20^, ^21^, ^22^ ^]^ Molecular functionality can modulate surface states within the silicon band gap, thereby affecting charge carrier injection across these interfaces. In molecularly modified metal–semiconductor junctions, this effect can alter charge transfer behavior, enabling a transition from diode‐like rectification to Ohmic conduction (similar to metals) depending on the molecular orientation and bonding.^[^ ^23^, ^24^ ^]^ A deeper understanding of this interplay is crucial for the design of molecular electronic devices with precisely controlled charge transfer properties.

Silicon surfaces, particularly hydrogen‐terminated silicon (Si─H), enable the formation of robust covalent bonds with alkenes, alkynes, thiols, hydroxyl groups, and other functionalities. These surface reactions lead to the formation of Si─C, Si─O, and Si─S bonds, which offer greater chemical and mechanical stability compared to metal–molecule linkages. Surface functionalization of Si─H can proceed via spontaneous reaction^[^ ^25^, ^26^, ^27^, ^28^, ^29^, ^30^ ^]^ or through the use of radical initiators,^[^ ^18^, ^31^, ^32^, ^33^, ^34^, ^35^, ^36^, ^37^, ^38^ ^]^ thus broadening the scope of molecular architectures that can be integrated with semiconductor materials. When multiple functional groups are present in a molecular system, their reactivity toward Si─H surfaces and the resulting molecular orientation can be harnessed to gain more control at the interface. For example, both hydroxyl (OH) and alkyne groups can react with Si─H surfaces, either spontaneously or under UV light.^[^ ^39^, ^40^, ^41^, ^42^ ^]^ However, it remains unclear which functional group reacts faster when both OH and alkyne groups are present on the same molecule, nor which group will dominate at the distal end of the resulting molecular film. Understanding the competition between different reactive groups and their impact on the electrical output of Si‐based electrochemical and electrical devices is vital for optimizing device performance.

**Figure 1:**
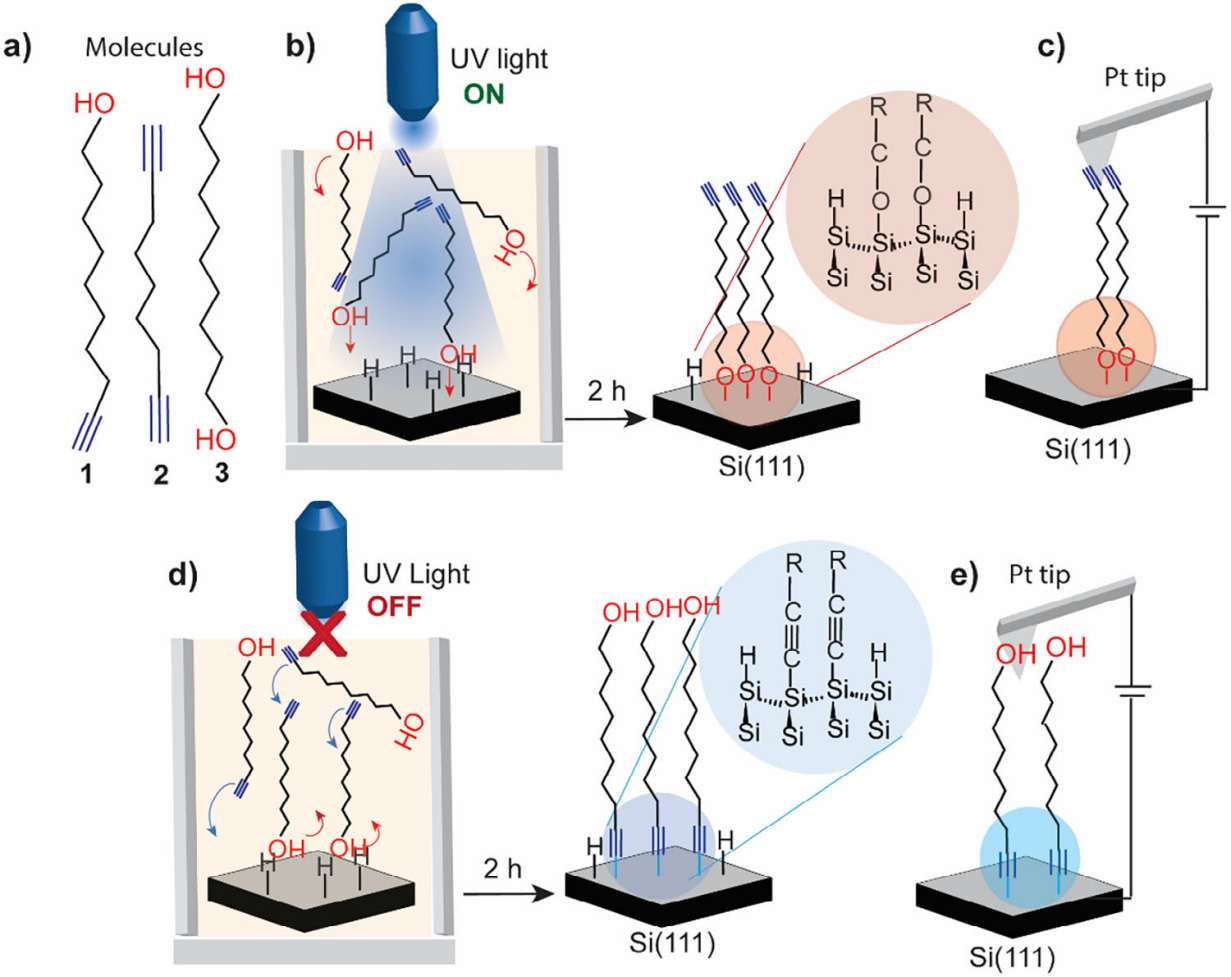
A schematic illustrating the chemical modification of Si─H surfaces and the control of the molecule–Si contact used in this study. a) Chemical structures of the investigated molecules (**1**–**3**). b) A schematic describing the UV‐assisted grafting of a monolayer of **1** through covalent Si─O─C bonding. c) Schematic representation of the C‐AFM measurements of Pt–**1**–Si junctions formed via UV‐assisted grafting of **1** on Si with a Pt AFM tip serving as the top electrode. d) A schematic illustrating the spontaneous grafting of a monolayer of **1** on Si─H via covalent Si─C bonding in the absence of UV light. e) A schematic representing the C‐AFM measurements of Pt–**1**–Si junctions formed via the spontaneous grafting of **1** on Si and a Pt AFM tip serving as the top electrode.

Here, we report metal–molecule–semiconductor junctions formed from molecules designed to position oxygen atoms either near the metal surface or near the semiconducting Si surface (**Figure** **1**). This was achieved by exploiting the competition between hydroxyl (OH) and alkyne functional groups in the same molecule to react with Si─H surfaces. We demonstrate that UV illumination significantly accelerates the reaction of OH groups with Si─H, forming Si─O─C linkages, while alkynes are more reactive in the absence of UV light, leading to Si─C linkages. This tunability in molecular orientation directly influences the rate of electron transfer in electrochemical measurements and the current in metal–semiconductor junctions. The current profiles of metal–molecule–semiconductor junctions were characterized using conducting atomic force microscopy (C‐AFM), while the surface properties of Si were analyzed through X‐ray photoelectron spectroscopy (XPS) and water contact angle goniometry. Electrochemical labeling of terminal alkyne groups with ferrocene via azide–alkyne click chemistry enabled the investigation of electrochemically driven electron transfer kinetics. By comparing electron transfer rate constants in molecular films with different orientations, we establish a correlation between molecular bonding with the electrode and charge transfer.

## 2 Results and Discussion

### 2.1 UV‐Light‐Controlled Molecular Orientation on Silicon

By taking advantage of the diversity of molecules that can be connected to Si and the ability of light to drive the reaction of two different functional groups (i.e., OH and alkyne) with Si─H surfaces, we can form metal‒molecule–semiconductor junctions with the molecule connected in two opposite orientations. The orientation of the molecules and hence current‒voltage responses from junctions formed with them are controlled by UV light, which accelerates the reaction with OH, whereas the reaction is sluggish with the alkyne. Both the OH and the alkyne groups can react with Si‒H to form Si─O and Si─C bonds, but at significantly different rates.

**Figure 2:**
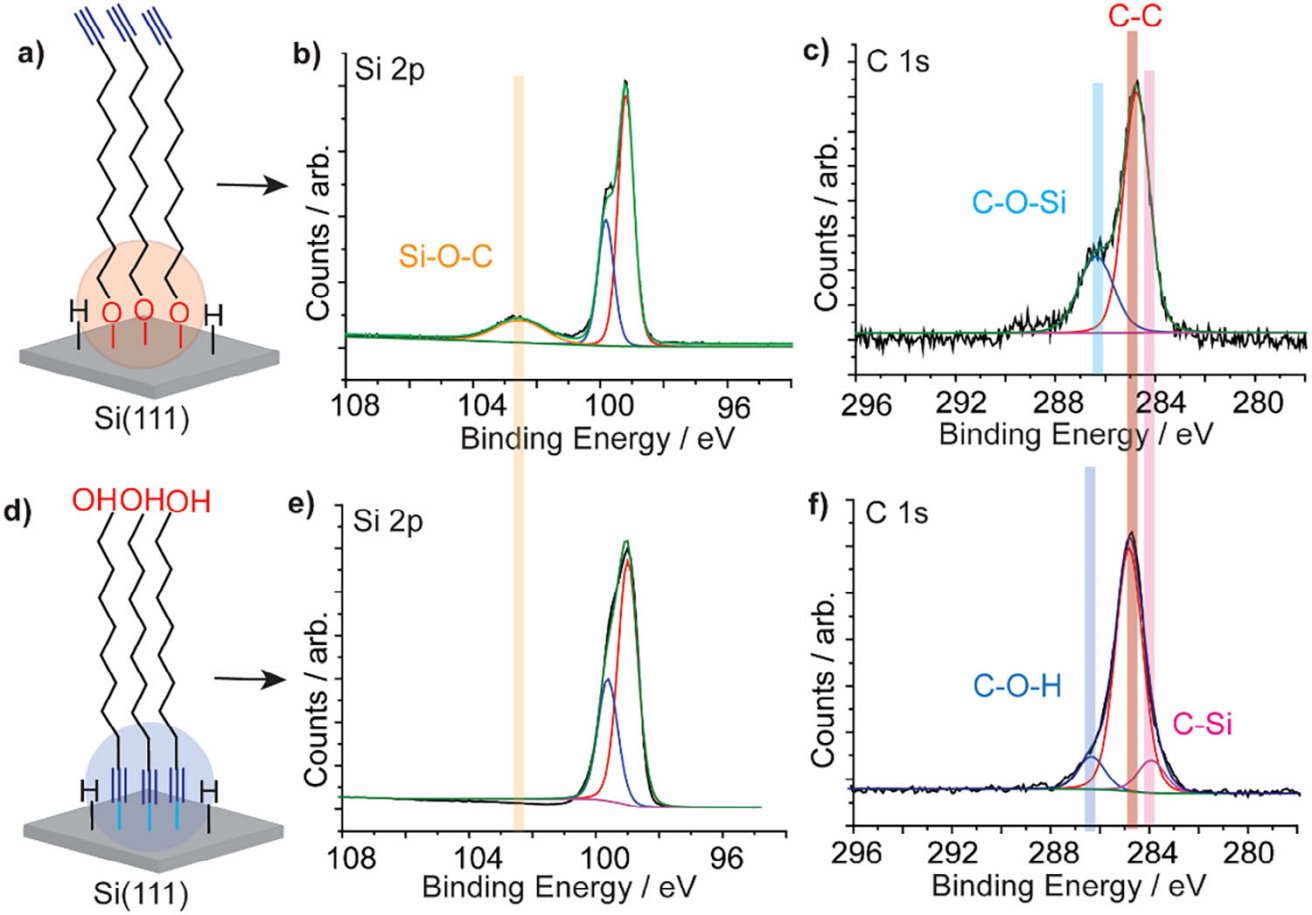
XPS analysis of a monolayer of **1** formed on Si─H surfaces either by UV‐assisted illumination or through spontaneous grafting. a) A schematic illustrating the type of chemical bonding at the molecule–electrode interface when the monolayer is formed via UV illumination. b) High‐resolution XPS Si 2p signal for the monolayer of **1** formed under UV illumination showing Si─O─C bonding at 103.6 eV in addition to a doublet at 99.3 eV corresponding to the Si spin‐orbit splitting. c) The C 1s spectrum for a monolayer of **1** formed by UV illumination shows two different carbon bonding states: emission at 284.7 eV corresponding to the C─C bonding and an emission peak at 286.3 eV corresponding to C─O─Si bonding. The absence of C‒Si signals at lower binding energy suggests that the molecule is bonded to the Si surface through its OH terminal. d) A schematic illustrating the chemical bonding when the monolayer of **1** is formed via spontaneous grafting. e) The Si 2p spectrum for the monolayer of **1** formed by spontaneous grafting shows the absence of the Si─O emission signal at ≈103 eV. f) The C 1s signal for the monolayer of **1** formed by spontaneous grafting shows three different carbon bonding states. The emission peaks at 284.0, 284.7, and 286.3 eV, correspond to C─Si, C─C, and C─OH bonding, respectively. This suggests that **1** reacts with the Si surface via its C≡C terminal, leaving the OH group free at the other distal end.

XPS analysis of monolayers formed from **1** by both UV‐assisted and spontaneous methods was performed to determine the molecule–electrode chemical bonding. **Figure** **2**a shows a schematic for the formed molecule–electrode chemical contact under UV light illumination. High‐resolution XPS spectra for Si 2p and the C 1s orbitals are shown in Figure 2b,c, respectively. The presence of Si─O emission at 103.6 eV in the Si 2p envelope is attributed to the formation of a covalent Si─O─C bond between **1** and the Si surface (Figure 2b). The C 1s spectrum revealed two emission signals at 284.7 eV and 286.3 eV corresponding to C─C and C─O─Si bonding, respectively (Figure 2c), demonstrating that **1** reacts with the Si surface via its OH terminal when illuminated by UV light. XPS survey spectrum shows all the elemental components expected for molecule **1** on Si (Figure S1, Supporting Information). Incubating a Si─H surface in ethanol for 2 hours showed no silicon oxide formation (Figure S2, Supporting Information), confirming the successful etching of the oxide and the relative stability of a bare, unfunctionalized Si─H surface during the course of the experiment.

XPS analysis of monolayers of **1** formed on Si−H via spontaneous grafting (Figure 2d) showed different chemical bonding to that fomed under UV light. The Si 2p envelope showed no signal for Si─O at ≈103 eV (Figure 2e). The C 1s spectra exhibited three emissions at 283.9, 284.7, and 286.2 eV, corresponding to the C─Si bond at the molecule–electrode interface, C─C of the alkyl chain, and the C─OH bonding, respectively (Figure 2f), suggesting that **1** binds to the Si─H surface via its Si‒C≡C contact. This is consistent with the spontaneous reaction of alkyne with Si**‒**H in the absence of UV light that has been reported by Buriak and co‐workers.^[^ ^43^, ^44^, ^45^ ^]^ Next, we analyzed the XPS spectra for films formed from **2,** which contains two terminal alkyne groups, and from **3**, that contains two OH groups at both ends.

Figure S3a (Supporting Information) shows high‐resolution Si 2p spectrum for **2** grafted spontaneously on Si─H, with no emission from Si‒O bonding expected at ≈103 eV. The C 1s emission in Figure S3b (Supporting Information) shows three peaks at 283.9, 284.7, and 289.1 eV, attributed to the C─Si, C─C/C≡C, and C═O bonding, respectively. Figure S4a (Supporting Information) shows the Si 2p emission for molecule **3** on Si grafted under UV light illumination for 2 h with a characteristic Si─O─C emission signal at 102.8 eV. The C 1s envelope in Figure S4b (Supporting Information) showed four emission signals at 284.7, 285.9, 286.6, and 288.7 eV, corresponding to the C─C, C─O─Si, C─O─H, and C═O bonding, respectively. These results are consistent with surface wettability measurements in Figure S5 (Supporting Information). Monolayers formed from molecule **1** under UV illumination showed a water contact angle of ≈81° suggesting the presence of a comparably less hydrophilic alkyne group at the distal end (Figure S5a, Supporting Information), while Si─H spontaneously functionalized with molecule **1** showed a contact angle of ≈34°, which is much more hydrophilic, confirming the presence of OH groups at the distal top end (Figure S5b, Supporting Information). In addition, contact angle measurements for Si─H surfaces functionalized with molecule **2** via spontaneous assembly showed a water contact angle of ≈84° (Figure S5c, Supporting Information), similar to the values measured for monolayers formed from **1** under UV illumination, and confirming the molecular orientation of **1** under UV illumination with the formation of Si─O bonds with the surface and terminal alkynes at the distal end. It should be noted that the reaction of Si─H with alkyne and hydroxyl (OH) groups is expected to generate H₂ gas. However, the amount of H₂ produced from a single‐molecule‐thick monolayer is likely too low to be observed as bubbles or detected using bulk analytical techniques. Density functional theory (DFT) calculations have previously shown that thiol (SH)‐terminated molecules, which are expected to react with Si─H surfaces in a manner analogous to OH, produce H₂ gas upon reaction with Si─H.^[^ ^26^ ^]^ However, this remains experimentally unverified. Nevertheless, several studies have reported the reaction of alkynes and hydroxyl groups with Si─H surfaces.^[^ ^39^, ^46^, ^47^ ^]^

**Figure 3:**
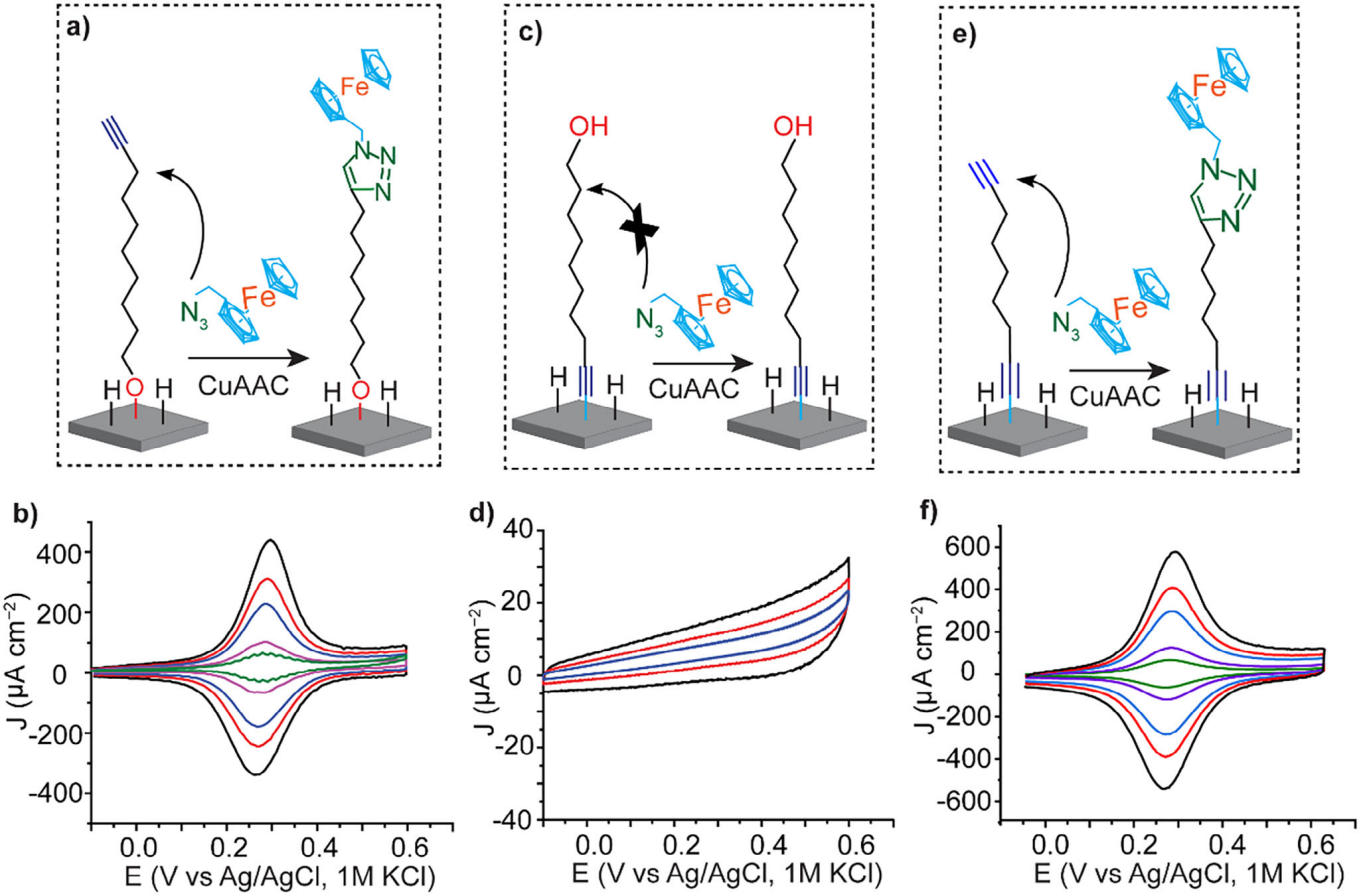
Electrochemical characterization of monolayers of **1** formed in the presence or absence of UV light, and of **2** formed via spontaneous assembly, followed by a CuAAC reaction with a ferrocene moiety. a) Schematic of the CuAAC reaction with the alkyne‐terminated monolayer formed from **1** by UV‐assisted grafting. b) The corresponding cyclic voltammograms for the Si─O─C enabled monolayer, displaying typical ferrocene redox waves at different scan rates (green: 1 Vs^−1^, purple: 2 Vs^−1^, blue: 5 Vs^−1^, red: 7 Vs^−1^ and black: 10 Vs^−1^). c) Schematic representing the CuAAC reaction with the monolayer formed from **1** by spontaneous grafting, enabling a Si─C molecule‒electrode contact and a terminal OH group. d) The corresponding cyclic voltammograms for the Si─C≡C bonded monolayers, showing the absence of the ferrocene redox signals at different scan rates (blue: 0.1 Vs^−1^, red: 0.2 Vs^−1^ and black: 0.5 Vs^−1^). e) Schematic of the surface functionalization of the alkyne‐terminated monolayer formed from **2** by spontaneous assembly, followed by a CuAAC reaction with a ferrocene moiety. f) Cyclic voltammograms for the Si─C≡C enabled molecular contact, showing characteristic ferrocene redox waves at different scan rates (green: 1Vs^−1^, purple: 2 Vs^−1^, blue: 5Vs^−1^, red: 7 Vs^−1^ and black: 10 Vs^−1^).

The Si─H electrodes that are functionalized with **1** in the presence and absence of UV illumination were also characterized electrochemically using cyclic voltammetry. Here, the monolayers react with an azidomethyl ferrocene on the surface via copper‐catalyzed alkyne−azide cycloaddition reaction (CuAAC).^[^ ^48^ ^]^ While the reaction is expected to be successful on the alkyne‐terminated monolayer, the reaction should not proceed with the OH‐terminated monolayer under the same conditions. **Figure** **3**a,b show a schematic for the CuAAC reaction with a monolayer of **1** formed on Si−H, under UV light illumination, and the corresponding cyclic voltammograms (CVs) at different scan rates. A typical ferrocene redox signal at 0.30 V (vs Ag/AgCl) was obtained with a surface coverage of 5.1 ± 0.3 10^−11^ mol cm^−2^ (Figure 3b). As a control experiment, CVs for a monolayer of **2** formed by spontaneous grafting (i.e., in the absence of UV light) showed a typical ferrocene redox signal with a surface coverage of 7.4 ± 0.5 10^−11^ mol cm^−2^ (Figure 3e,f). The evolution of the anodic and cathodic peak currents scales linearly with the scan rate, indicating that the redox reaction represents a surface‐bound redox system (Figure S6a, Supporting Information). In addition, successive voltametric cycling showed negligible decrease in the peak current, demonstrating the high stability of the Si─O─C bound monolayers (Figure S6b, Supporting Information). Conversely, CVs of Si−H electrodes functionalized with **1** via the spontaneous assembly method enabled the formation of Si─C─ bonding at the molecule–electrode interface, with the absence of the ferrocene redox signals after the CuAAC reaction with azidomethyl ferrocene. These results demonstrate that in the absence of UV light, **1** reacts with the Si─H surface via Si─C≡ bonds, and the OH moieties are now at the distal end of the film (Figure 2d). As a control experiment, CVs of a monolayer of **2** formed by spontaneous grafting (i.e., in the absence of UV light) showed the typical ferrocene redox signals (Figure 3e,f), demonstrating that alkynes can indeed react with Si─H surfaces in absence of UV light. On the other hand, a monolayer of **3** formed by UV light illumination followed by a CuAAC reaction showed no ferrocene redox signals (Figure S7, Supporting Information), demonstrating that no reaction occurred.

### 2.2 Interfacial Bonding Effects on Molecular Circuit Output

**Figure 4:**
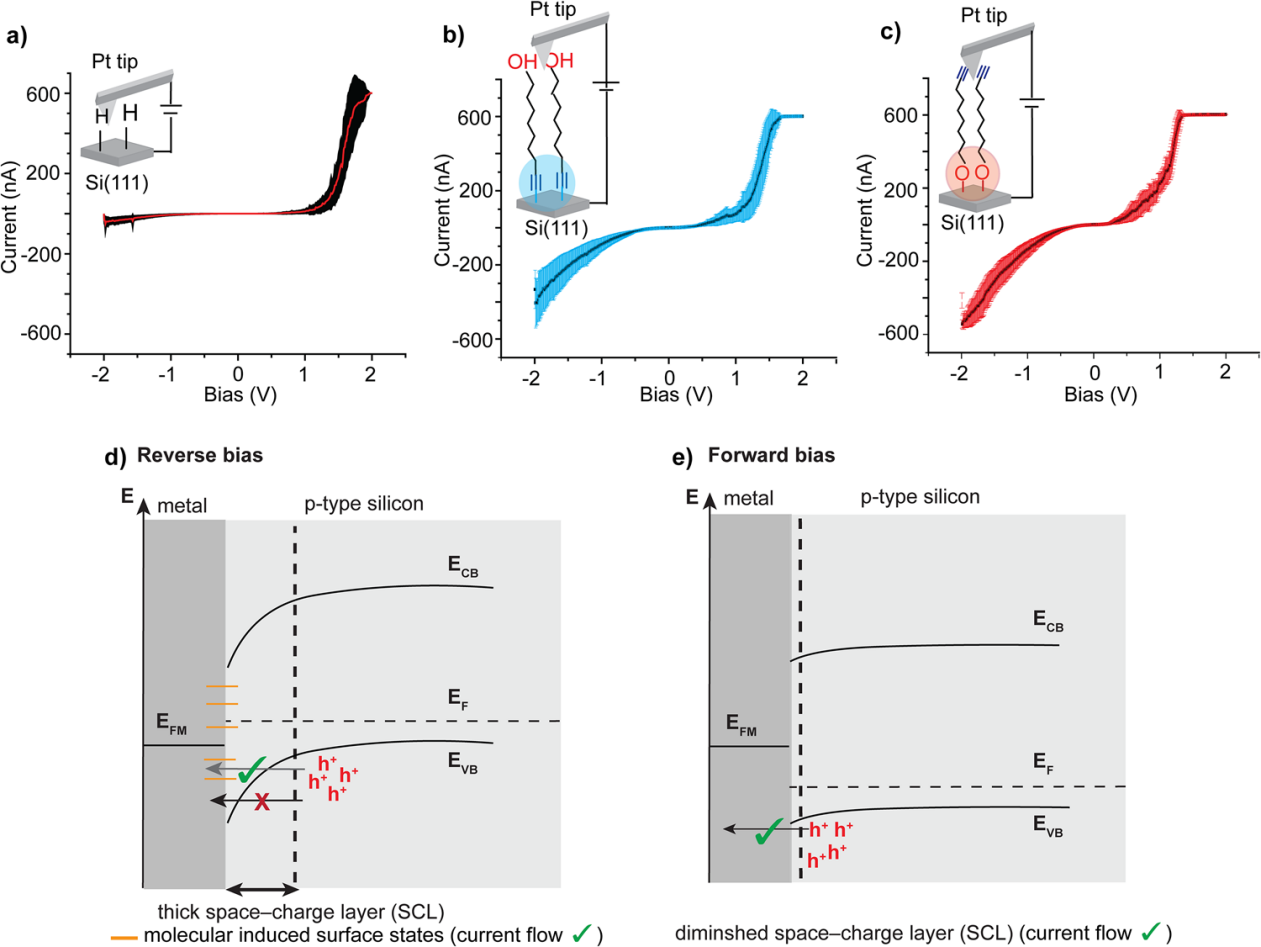
a) *I–V* characteristics of a platinum AFM tip in contact with a freshly etched p‐type Si─H surface (Pt–Si junction). b) *I–V* characteristics of Pt–**1**–Si junctions formed via spontaneous monolayer formation of **1**, creating a Si‒C≡C interface. c) *I–V* characteristics of Pt–**1**–Si junctions formed by UV‐assisted grafting of **1**, creating a Si─O─C interface. d) Schematic energy band diagram of the Pt–Si junction under reverse bias, showing a wide space‐charge layer (SCL) that inhibits current flow. In Pt–**1**–Si junctions, monolayer‐induced surface states reduce band bending and thin the SCL, allowing increased current. e) Schematic energy band diagram of the Pt–Si interface under forward bias, where the system enters accumulation and the SCL is diminished, enabling current flow regardless of the molecular layer between the metal and silicon. The solid line in a–c) represents the average of 40 different measurements. E_FM_ and E_F_ represent the Fermi levels of the metal and Si, respectively, while E_CB_ and E_VB_ denote the energies of the conduction band and valence band edges of Si, respectively.

The effect of tuning the molecule‐electrode contact on the electrical properties of the metal‒molecule‒semiconductor junctions was studied using conductive atomic force microscopy (C‐AFM). **Figure** **4**a shows the current–voltage (*I‒V*) measurements for Pt‒Si(111)‒H p‐type junctions, revealing a current rectification of ≈200 at ± 1.5 V bias.

Figure 4b shows the *I‒V* measurements for a Si surface functionalized with **1** via spontaneous assembly where the Si─C≡ contact dominates the molecule–electrode interface (Pt–**1**–Si junctions). Both the forward and reverse currents increase relative to unmodified Si─H, but with notably higher reverse currents (≈400 nA). The *I–V* characteristic of the Si surface functionalized under UV light illumination (leading to Si─O─C contact), showed a larger increase in both the forward and reverse currents (≈600 nA), with current saturation observed at both bias polarities (Figure 4c). These results indicate that introducing a molecule between metals and semiconductors of Schottky diodes alters the current‒voltage characteristics from a rectifier that passes current only in one direction to a resistor‐like behavior, enabling current flow in both bias directions. The junctions formed via Si─O─C bonding exhibit higher conductivity than those formed via Si─C bonding. For a p‐type Si that is under accumulation condition, as in the case studied here, the majority charge carriers (h^+^) will flow between the Si and the Pt tip when a positive bias is applied to the Si. However, the current flow is limited under negative bias due to the system entering depletion, which is accompanied by the formation of a thick space charge layer (SCL), beyond the electron tunnelling length (≈1 nm) (Figure 4c).^[^ ^49^, ^50^, ^51^, ^52^ ^]^

Figure 4d,e shows the energy band diagram of the Si‒Pt interface under reverse bias (Figure 4d) and under forward bias (Figure 4e). Under reverse bias, the system is under depletion; therefore, the SCL is thick and the energy bands in the p‐type Si bend downward, preventing current flow. Introducing molecules between Pt and Si appears to flatten the bands and thin the SCL, which could be due to one of two scenarios: 1) the presence of a molecular dipole moment that decreases the SCL thickness or 2) the creation of surface states as new energy levels within the Si band gap that allow for charge transfer. If the interfacial dipole created by the molecular bonding is the dominant effect, it is expected that the closer the electronegative oxygen atom to the Si surface, the more likely the holes (h^+^) in the p‐type Si are pushed away from the interface (Figure S8, Supporting Information), resulting in a thicker depleting region (SCL). However, since current is observed to flow in both directions, it is likely that surface dipole effects are not dominant; rather, the second scenario—surface state formation—is more plausible. We therefore hypothesize that inserting an oxygen‐containing molecule between Pt and p‐type Si contact creates negative surface states^[^ ^53^, ^54^ ^]^ that attract holes, therefore diminishing the SCL. Hence, the closer the oxygen atom is to the Si surface, the smaller the SCL, and the more conductive the junction becomes under both bias polarities. By strategically placing oxygen either close to or away from the Si surface, the junction properties can be tuned.

### 2.3 Kinetics of Electrochemical Electron Transfer

**Figure 5:**
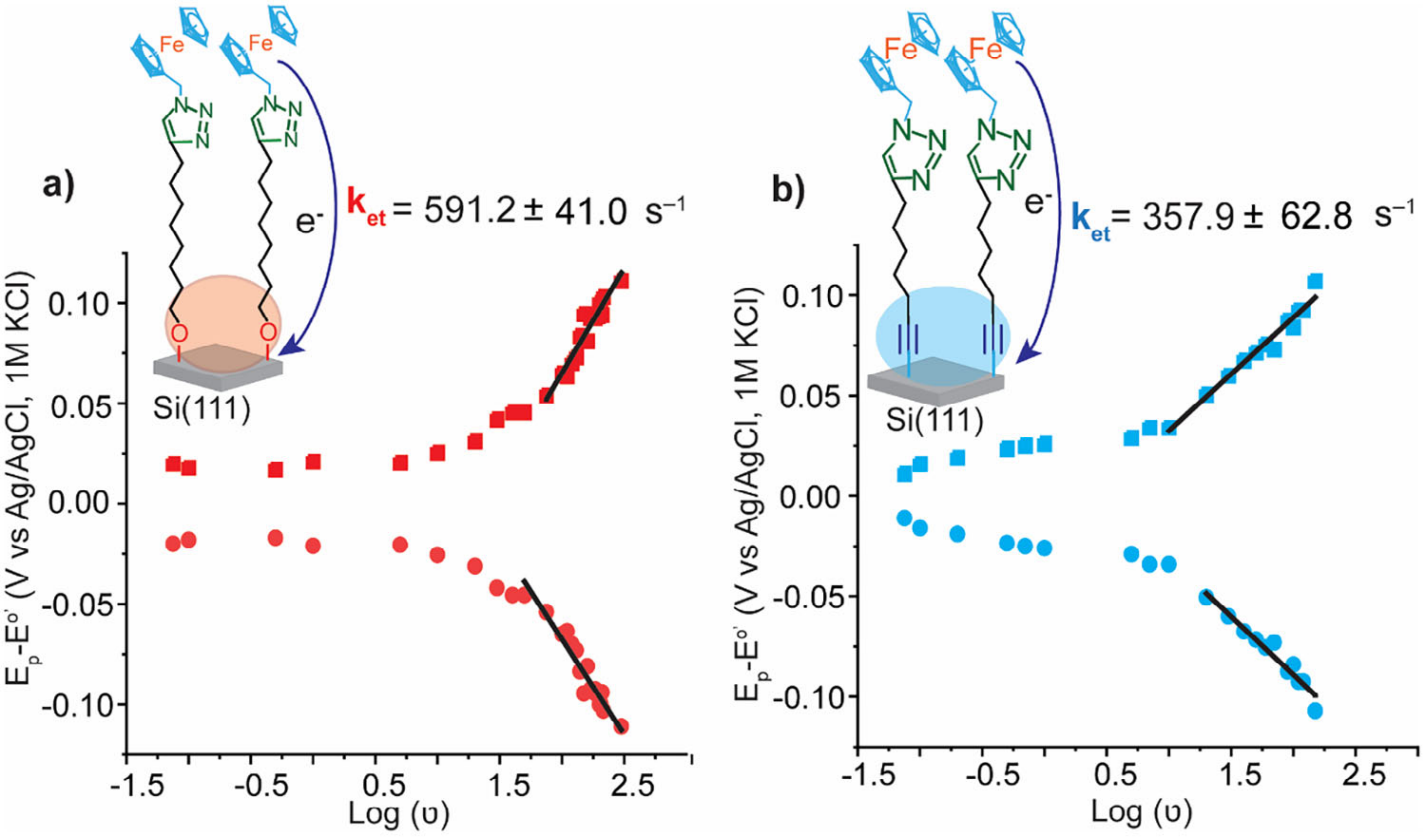
a) The evolution of the peak potential minus the apparent formal potential (E_p_–E^0’^) versus the logarithm of the scan rates for the Si─O─C contact‐enabled monolayers, with calculated *k*_et_ of 591 ± 41 s^−1^. b) The evolution of the peak potential minus the apparent formal potential (E_p_–E^0’^) for the Si─C contact‐enabled monolayers with calculated *k*_et_ values of 358 ± 62 s^−1^. The values of E^0’^ were obtained from cyclic voltammograms at slow scan rates.

The correlation between the conductance under dry conditions ‒ in large area and single‒molecule electronics ‒ and charge transfer rates of electrified solid–liquid interfaces, represented by *k*_et_, has been debated among theoreticians and experimentalists.^[^ ^55^ ^]^ The correlation of *k*_et_ with the conductance of a surface‐bound redox molecular film is controversial, as *k*_et_ is influenced not only by charge transfer but also by reorganization energy—the energy required to reorganize the molecular and solvent environment during charge transfer.^[^ ^56^ ^]^ To further investigate the impact of controlling the molecule–electrode contact on electrified interfaces, and whether it affects *k*_et_ in the same way it influences currents measured in nanoscale junctions, we calculated the *k*_et_ for a ferrocene‐terminated monolayer of **1** in a three‐electrode electrochemical cell. The evolution of the peak potentials for the oxidation and reduction waves of the ferrocene moieties in cyclic voltammetry was found to depend on the type of molecule–electrode bond: Si‒O‒C contact versus Si‒C≡ contacts, suggesting a different *k*_et_. The scan rate dependence of the peak potentials was analyzed using the Laviron method to extract *k*_et_ values.^[^ ^57^, ^58^ ^]^ When the molecule was bound to the electrode via the Si─O─C bond, the *k*_et_ value was ≈ 591 ± 41 s^−1^, whereas when the molecule was connected via Si─C≡C bond, the *k*_et_ was ≈358 ± 62 s^−1^ (**Figure** **5**a,b). To investigate the influence of surface coverage on electron transfer kinetics, we compared the monolayers formed from molecules **1** and **2**, which exhibit surface coverages of 5.1 ± 0.3 × 10⁻¹¹ and 7.4 ± 0.5 × 10⁻¹¹ mol cm⁻^2^, respectively. Although previous studies have reported that higher surface coverage in ferrocene‐terminated monolayers enhances electron transfer rates^[^ ^18^, ^59^, ^60^ ^]^ our results show the opposite trend: monolayer **2**, despite its slightly higher surface coverage, exhibits slower electron transfer kinetics, with a rate constant nearly half that of monolayer **1**. This suggests that the observed differences in electron transfer kinetics and C‐AFM currents are primarily governed by the nature of the molecule–electrode bond rather than the density of surface‐bound ferrocene moieties. These findings highlight that bond formation at the Si surface plays a more significant role in modulating charge transfer than variations in molecular packing density. It is also worth noting that molecule **1** contains two additional C─C bonds compared to molecule **2**. Hence, one would expect the *k*_et_ values for molecule **1** to be lower due to the increased molecular length, as predicted by the distance‐dependent attenuation factor (β) of 1 bond^−1^ for saturated molecules. However, the measured *k*_et_ values for molecule **1** were found to be twice as high as those for molecule **2**. This discrepancy suggests that oxygen atoms directly connected to Si play a more significant role in determining the charge transfer  efficiency, as they reduce the space‐charge region, allowing for more efficient charge conduction despite the extra C─C bonds in molecule **1**. This observation is also consistent with the C‐AFM measurements, which recorded higher currents for Si–**1**–Pt junctions compared to Si–**2**–Pt junctions.

## 3 Conclusion

In summary, UV light was used to control both the reaction and the orientation of an organic molecule at metal–Si junctions, as well as between Si and a surface‐bound redox moiety. The key molecule used (**1**) features an OH group at one end and an alkyne group at the other. UV illumination facilitated reaction through Si─O─C bonds, while in the absence of UV light, bonding occurred via the alkyne terminal group, resulting in Si─C bonds as characterized by XPS and water contact angle measurements. The results show that under UV light, OH groups react with Si─H surfaces much faster than alkynes, resulting in surface termination with alkyne moieties. While OH is more reactive to Si─H than alkynes under UV light, alkynes are more reactive than OH in the absence of UV light, where the reaction occurs spontaneously.

In both metal–molecule–Si junctions measured using C‐AFM and Si–redox‐molecule–electrolyte configuration studied electrochemically, electrode–molecule contacts facilitated by Si─O─C bonds exhibited a conductance and an electrochemical electron transfer rate constant twice as high as those formed via Si─C bonds, despite being two C─C bonds longer. The enhanced conductance and charge transfer kinetics of Si─O─C‐modified Si, compared to Si─C, are attributed to surface states induced by the proximity of oxygen to the Si surface. This study demonstrates the impact of atomic‐level positioning of molecular surface states on charge transfer at the Si–molecule–metal and silicon–electrolyte electrified interfaces. By precisely controlling the placement of oxygen atoms—either directly on the Si surface or near the metal or the electrolyte side—we introduce molecular surface states that significantly influence the space charge layer. This level of fine‐tuning offers a new approach to modulating the properties of Si‐based electrical and electrochemical devices.

## 4 Experimental Section

### Chemicals

All chemicals were purchased from Sigma‐Aldrich unless otherwise specified. Sulfuric acid (Puranal TM, 95−97%), hydrogen peroxide (30 wt.% in water) 9–decyn–1–ol (94%) was purchased from Fisher Scientific, USA. 1, 8–nonadiyne (98%) and 1, 9–nonanediol (98%) were purchased from Sigma Aldrich and used without further modification. The solvents, including dichloromethane (DCM), acetonitrile, isopropyl alcohol, are distilled before use. P‐type highly boron‐doped silicon wafers with resistivity of 0.007 Ω cm and thickness 500 ± 25 µm oriented ± 0.5° away from the (111) plane were purchased from Siltronix, S.A.S. (Archamps, France). Azidomethyl ferrocene was synthesized following the procedure of Ciampi et al.^[^ ^22^ ^]^

### Surface and Monolayers Preparations

Si electrodes were cut from a wafer (≈1 cm^2^), then treated with piranha solution, a mixture of concentrated sulfuric acid and 30% hydrogen peroxide, 3:1 (v:v), respectively for 30 min. The electrodes were then extensively rinsed with Milli‐Q water and etched with a deoxygenated aqueous ammonium fluoride solution (40%) for 13 min, washed with Milli‐Q water and DCM then stored in deoxygenated DCM before further analysis. For UV light‐assisted grafting, the vial that contains molecule **1** or **3** was placed inside an air‐tight, light‐proof reaction chamber equipped with a UV light source. The chamber was kept under positive Ar gas pressure. Molecules **1** and **3** were dissolved in ethanol (≥99.5%, anhydrous) and the solution was bubbled with Ar gas for 10 min before immersing the freshly etched Si─H surface inside the solution with or without UV light. The freshly etched Si─H electrode was then transferred with tweezers into the vial while the UV light was switched on. The vial was then sealed and exposed to UV illumination for 2 hours. The UV light was provided by a collimated LED source (λ  =  365 nm, nominal power output >190 mW, Thorlabs part M365L2 coupled to a SM1P25‐A collimator adapter), and was fixed over the sample at a distance of ≈10 cm. After illumination, the functionalized Si electrodes were rinsed with DCM and dried with a stream of Ar gas before further use. For the spontaneous grafting method, the Si─H electrodes were immersed in a vial containing either molecule **1** or **2** and kept in the dark for 2 hours, then washed with DCM and dried under Ar gas before further use. For the spontaneous monolayer formation, the duration of the reaction was optimized by carrying out the assembly at different times with the maximum coverage achieved within 2 hours.

### XPS Analysis

XPS measurements were performed on a Kratos Axis Ultra DLD spectrometer, using a monochromatic Al‐Kα (1486.6 eV) irradiation source operating at 150 W. Spectra were acquired in normal emission at or below 7 × 10^−9^ Torr. Data files were processed using CasaXPS software, applying Shirley background subtraction. All binding energies were aligned by applying a rigid shift to bring the C 1s peak to 284.7 eV.

### Electrochemical Characterization

The monolayer functionalized Si electrodes were rinsed with DCM and 2‐propanol. The Si electrodes were then transferred into a reaction tube containing 2 mL of azidomethyl ferrocene solution (0.5 mm, 2‐propanol/water, 1:1), 2 mL of copper (II) sulfate pentahydrate (5 mg mL^−1^) and 1 mL sodium ascorbate (0.4 mm), and the CuAAC reaction was carried out at room temperature in the dark for 1 h.^[^ ^48^ ^]^ The reaction was then stopped by removing the ferrocene‐functionalized electrodes from the reaction tube followed by sequential washing with copious amounts of 2‐propanol, water, 0.5 m aqueous hydrochloric acid, water, 2‐propanol, and DCM. The functionalized surfaces were then kept in ethylenediaminetetraacetic acid EDTA (0.05% w/v, pH 7.4) for 2 h to chelate any residual copper, before rinsing with water and drying with a stream of Ar gas before further analysis. All electrochemical measurements were performed in a single‐compartment, three electrode PTFE cell using an electrochemical workstation, CHI650 (CH Instruments, USA). A platinum wire served as the counter electrode, Ag/AgCl aqueous electrode (1 m KCl) as the reference electrode and the functionalized Si electrode as the working electrode. The electrolyte used was an aqueous solution of 1 m NaClO_4_.

### AFM Measurements

Conductive AFM I‒V measurements were carried out in air and at room temperature using conductive platinum tips (Rocky Mountain Nanotechnology AFM probes, 25Pt300B, with a spring constant of 18 N m^−1^). The imaging resolution was set to 256 points/line and a scan rate of 1 Hz. The *I‒V* measurements were performed at voltage sweep rates of 2 V s^−1^ and with the feedback being switched to contact mode with the deflection setpoint kept constant to 500 nN. The deflection setpoint corresponding to 500 nN was selected to ensure that the measured current was independent of the applied force.

## Conflict of Interest

The authors declare no conflict of interest.

## Supporting information



Supporting Information

## Data Availability

The data that support the findings of this study are available from the corresponding author upon reasonable request.
