# Supplementary material for: Tunable Nanoscale Metal‒Molecule‒Semiconductor Junctions via Light‐Controlled Molecular Orientation
Source: Small. 2025 May 19;21(29):2412438. doi: 10.1002/smll.202412438 (PMC12288777; doi:10.1002/smll.202412438)
Supplement: Supplementary file 1 — Supporting Information [file SMLL-21-2412438-s001.pdf]

NANO · MICRO
small

Supporting Information

for *Small*, DOI 10.1002/smll.202412438

Tunable Nanoscale Metal–Molecule–Semiconductor Junctions via Light-Controlled Molecular Orientation

*Essam Mohamed Dief, Tiexin Li, Ingrid Ponce and Nadim Darwish**

Supporting Information

Tunable Nanoscale Metal–Molecule–Semiconductor Junctions via Light-Controlled Molecular Orientation

Essam M. Dief[1], Tiexin Li[1], Ingrid Ponce[2] and Nadim Darwish[1]*

[1]School of Molecular and Life Sciences, Curtin University, Bentley, WA 6102, Australia

[2]Departamento de Ciencias del Ambiente, Facultad de Química y Biología, Universidad de Santiago de Chile, Av. Libertador Bernardo O'Higgins 3363, Estación Central, Santiago, Chile

Corresponding author: nadim.darwish@curtin.edu.au

KEYWORDS: Metal–semiconductor junctions, molecule–electrode contacts, molecular switches, silicon, molecular electronics.

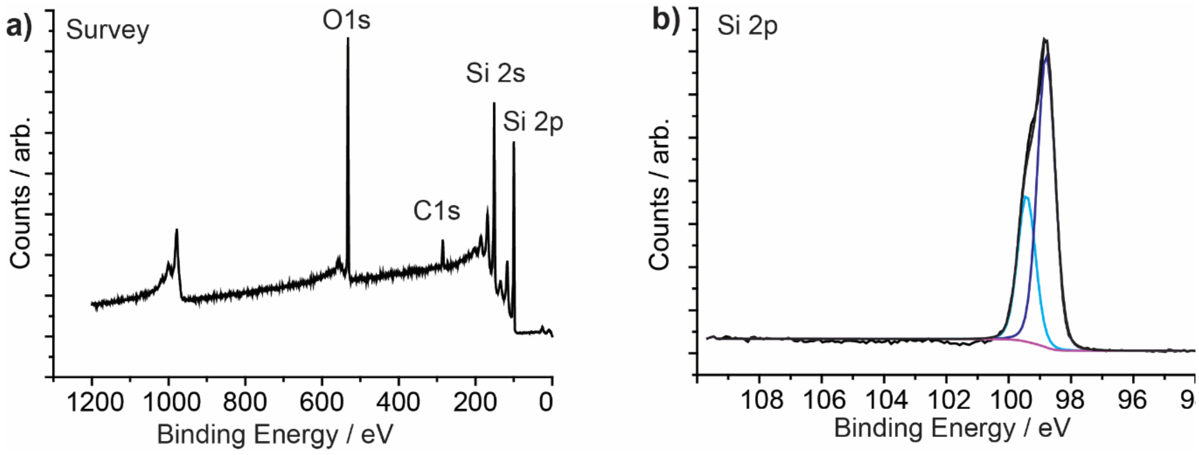

Figure S1. a) XPS survey spectrum for a monolayer of **1** formed on a Si–H surface by UV illumination for 2 h. b) High-resolution Si 2p spectrum for a freshly prepared Si–H surface kept in DCM solvent for 2 h, showing no oxide formation at ~103 eV.

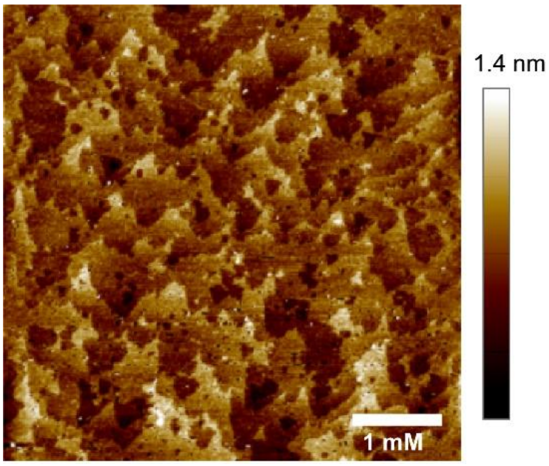

Figure S2. AFM topography image of a freshly etched Si–H surface that was incubated for 2 h in ethanol showing no significant oxidation occurring within this timescale.

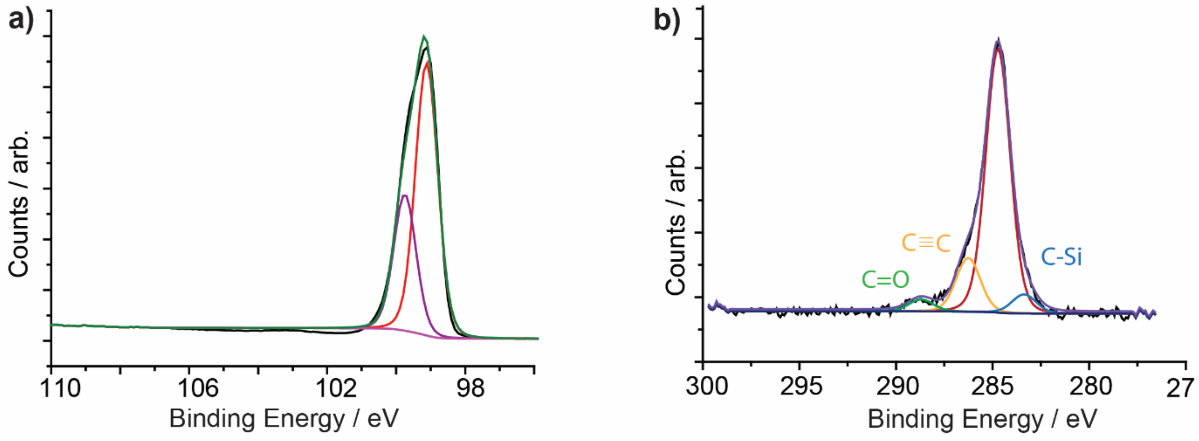

Figure S3. a) High-resolution Si 2p spectrum for a monolayer of **2** formed on Si via spontaneous grafting for 2 h. b) The C 1s signal for a monolayer of **2** formed on a Si surface by spontaneous grafting. The emission peaks at 283.9 eV, 284.7 eV, and 289.1eV are attributed to the C–Si, C–C/C≡C, and C=O bonding, respectively.

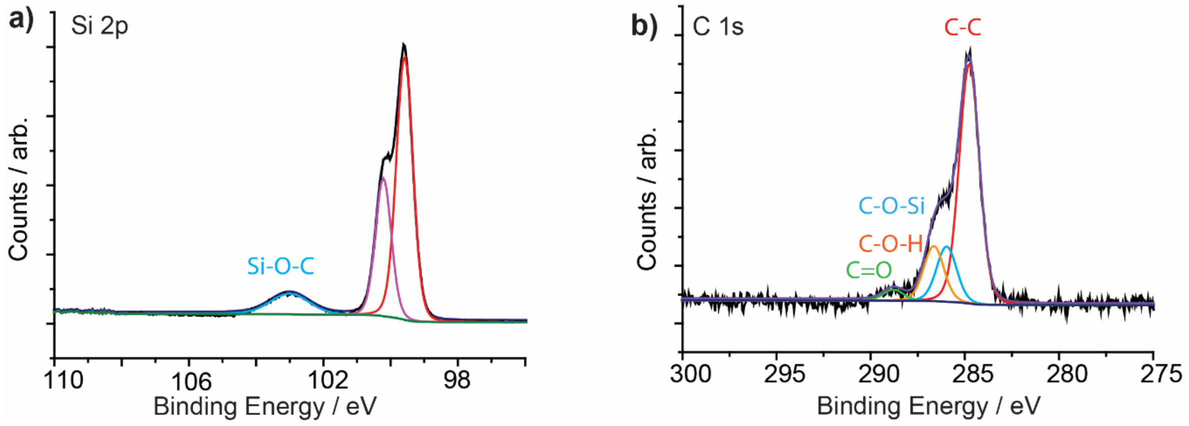

Figure S4. a) High-resolution Si 2p spectrum for a monolayer of **3** formed on Si via UV illumination for 2 h, showing the core Si–Si bonding at 99.3 eV and the Si–O–C bonding at 103.6 eV. b) High-resolution C 1s spectrum of a monolayer of **3** formed on a Si surface under UV illumination showing emission peaks at 284.7 eV, 285.9 eV, 286.6, and 288.7 eV, corresponding to the C–C, C–O–Si, C–O–H, and C=O bonding, respectively.

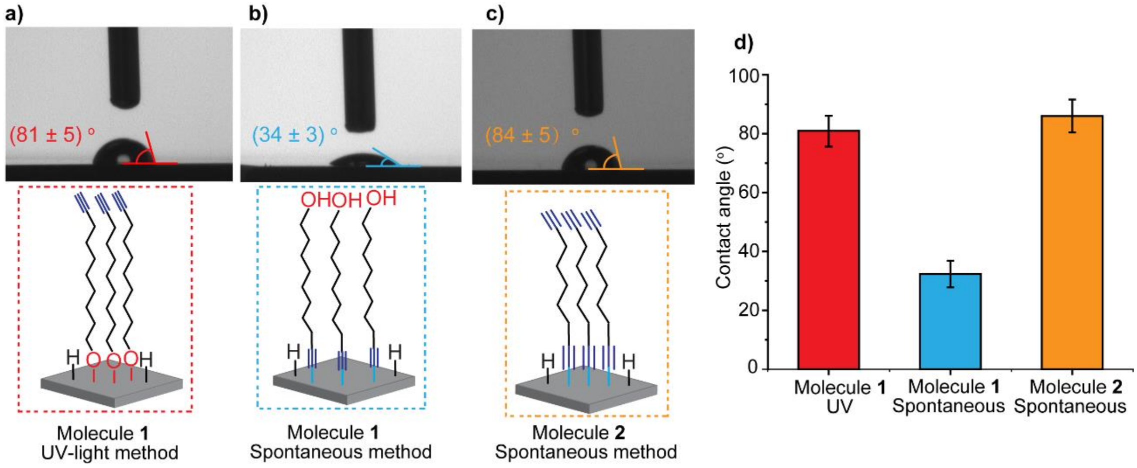

Figure S5. Water contact angles for a Si–H surface functionalised with molecule **1** under UV illumination (a) and spontaneously (b). Water contact angles for a Si–H surface functionalised with molecule **2** using the spontaneous method (c). d) Bar chart depicting the average water contact angles for the surfaces in (a–c) with the error bars representing the standard deviation of three different samples.

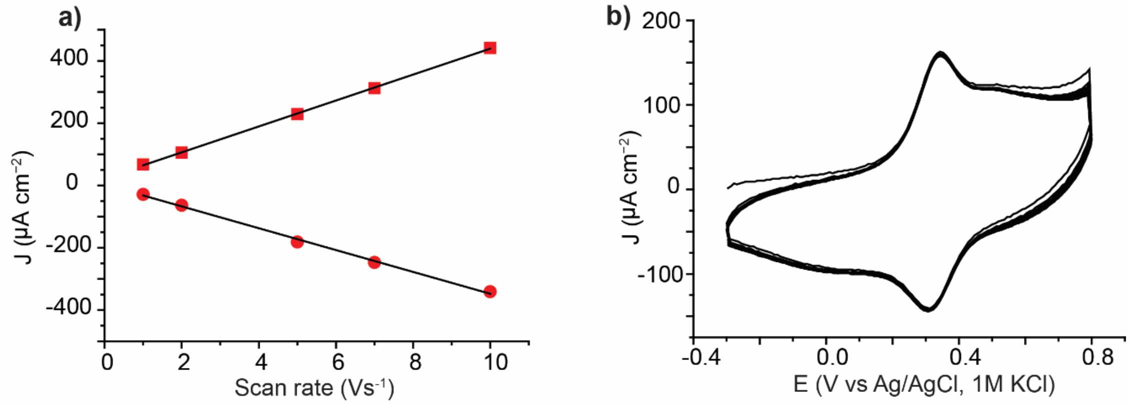

Figure S6. a) Peak current density versus scan rates for a monolayer of **1** formed on Si−H by UV illumination for 2 h, showing a linear relationship between the scan rate and the peak current, indicating a surface-bound redox reaction. Dots are data points and lines are the linear fitting. b) Repetitive cyclic voltammograms for a monolayer of **1** on Si−H surfaces at a scan rate of 2 Vs^{-1}.

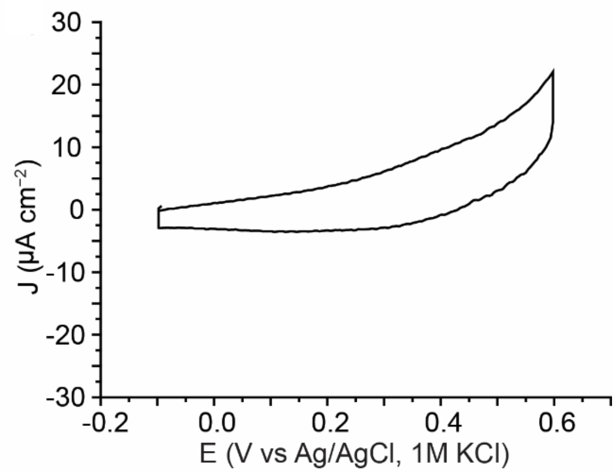

Figure S7. Cyclic voltammograms for a monolayer of **3** formed by spontaneous grafting on a Si–H surface for 2 h, followed by a CuAAC reaction with azidomethyl ferrocene for 1 h. The monolayer does not show ferrocene redox signals, indicating that only the alkyne groups can react with azidomethyl ferrocene via CuAAC reaction and not OH groups as is the case with **3**.

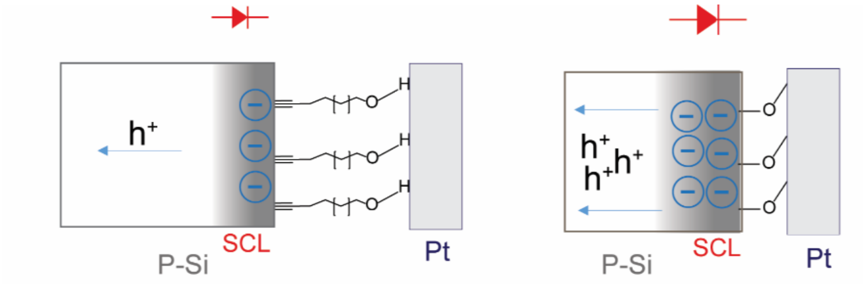

Figure S8. Schematic illustrating the unlikely scenario in which surface molecular dipole effects are responsible for converting the junction from rectifying to Ohmic behaviour. If the effect is driven by the molecular dipole, then the closer the electronegative oxygen atom is to the silicon surface, the more effectively it can repel holes (h^+), leading to the formation of a thicker depletion region (space charge layer, SCL), represented as a grey zone containing negatively charged ionized dopants. This scenario should lead to a more rectifying junction when the molecule is inserted between the metal and the semiconductor. Therefore, we conclude that the introduction of surface states upon the reaction of molecules between platinum and silicon is the main reason for the observed transition from rectifying to Ohmic characteristics (Figure 4 main manuscript).
